# Evaluating the Utility of a Structured Clinical Protocol for Reducing the Impact of Behavioural and Psychological Symptoms of Dementia in Progressive Neurological Diseases: A Pilot Study

**DOI:** 10.1155/2018/5420531

**Published:** 2018-03-26

**Authors:** Nicholas P. Ryan, Laura Scott, Maryanne McPhee, Susan Mathers, Marie-Claire Davis, Roxanne Maule, Fiona Fisher

**Affiliations:** ^1^Calvary Health Care Bethlehem, South Caulfield, VIC, Australia; ^2^Melbourne School of Psychological Sciences, The University of Melbourne, Parkville, VIC, Australia

## Abstract

**Objectives:**

Behavioural and psychological symptoms of dementia (BPSD) cause significant distress to both aged care residents and staff. Despite the high prevalence of BPSD in progressive neurological diseases (PNDs) such as multiple sclerosis, Huntington's disease, and Parkinson's disease, the utility of a structured clinical protocol for reducing BPSD has not been systematically evaluated in PND populations.

**Method:**

Staff (*n* = 51) and individuals with a diagnosis of PND (*n* = 13) were recruited into the study, which aimed to evaluate the efficacy of a PND-specific structured clinical protocol for reducing the impact of BPSD in residential aged care (RAC) and specialist disability accommodation (SDA) facilities. Staff were trained in the clinical protocol through face-to-face workshops, which were followed by 9 weeks of intensive clinical supervision to a subset of staff (“behaviour champions”). Staff and resident outcome measures were administered preintervention and immediately following the intervention. The primary outcome was frequency and severity of BPSD, measured using the Neuropsychiatric Inventory-Nursing Home Version (NPI-NH). The secondary outcome was staff coping assessed using the Strain in Dementia Care Scale (SDCS).

**Results:**

In SDA, significant reductions in staff ratings of job-related stress were observed alongside a statistically significant decrease in BPSD from T1 to T2. In RAC, there was no significant time effect for BPSD or staff coping; however, a medium effect size was observed for staff job stress.

**Conclusions:**

Staff training and clinical support in the use of a structured clinical protocol for managing BPSD were linked to reductions in staff job stress, which may in turn increase staff capacity to identify indicators of resident distress and respond accordingly. Site variation in outcomes may relate to organisational and workforce-level barriers that may be unique to the RAC context and should be systematically addressed in future RCT studies of larger PND samples.

## 1. Introduction

Behavioural and psychological symptoms of dementia (BPSD) represent a significant challenge in the clinical care and management of individuals living with progressive neurological diseases (PNDs). About 300 per 100,000 people are affected by the two most common conditions in this group: multiple sclerosis (MS) and idiopathic Parkinson's disease (PD). A further significant proportion of people are affected by Huntington's disease (6–14 per 100,000) and motor neurone disease (4–8 per 100,000) [1]. These conditions generally affect individuals from early to late midlife and are associated with complex patterns of physical, cognitive, and behavioural impairment [2].

Though the nature and correlates of BPSD in PNDs are not well characterised, challenging behaviours such as aggression, irritability, shouting, repetitive questions, and sexual disinhibition are common and may underlie high rates of carer distress and burnout in these populations [3]. Not surprisingly, BPSD represent one of several factors that prompt a decision to move individuals with PNDs into residential facilities, where prevalence of BPSD is also high [4, 5].

In the Australian context, BPSD are linked to stress and burnout among nursing care staff [6], many of whom are poorly paid and lack specialist skills required to effectively manage these behaviours [7]. Specifically, research shows that inadequate staff training is associated with staff stress and burnout, which in turn increases risk for resident BPSD [8, 9].

Additionally, BPSD are costly and resource intensive. For instance, these symptoms typically prompt referrals to external support agencies and represent a leading cause of emergency calls to mental health services [10]. Owing to the substantial psychosocial and financial impact of BPSD, evidence-based protocols to reduce the impact of BPSD are required, particularly among PND populations where the evidence base for nonpharmacological interventions is scarce [11, 12].

BPSD in individuals with PNDs represent a complex multidimensional construct, likely reflecting an interplay of aetiological factors that precipitate and maintain symptoms. The complexity of BPSD is reflected in recent international guidelines (e.g., International Psychogeriatric Association Complete Guide to BPSD [13]), which delineate multiple aetiologies for BPSD, including genetic, psychosocial, neurobiological, medical, and physical factors which interact dynamically to give rise to challenging behaviours. This dynamic interplay of variables is particularly salient for individuals living with progressive neurological diseases, for whom the onset of symptoms occurs relatively early in life.

Although BPSDs may present in a superficially similar way across patients, the complex mix of causal factors underlying BPSDs in PND populations means that there is substantial variability in the specific factors that trigger and perpetuate BPSDs within the individual patient [14]. In keeping with this model, it follows that addressing case-specific causal factors (i.e., using a personalized individual approach) would be a critical component of an evidence-based treatment model for BPSD. That is, while genetic and neurobiological variables may not be currently treatable, psychosocial and acute/subacute physical and medical factors are potentially malleable to nonpharmacological interventions. For instance, factors such as pain or depression [15], loneliness [16], cognitive impairment [17], sensory impairment [18], overstimulation [19], and even the way personal care is carried out [20] are all potential contributing factors to BPSD in PNDs. All these variables represent modifiable targets for individualized, person-centred interventions that aim at addressing treatable causes of resident suffering. In keeping with this biopsychosocial perspective, Brechin et al. [21] argue for a stepped-care model of assessment and intervention, which centres on a case-specific approach to determine the factors related to BPSD, and implement appropriate interventions, primarily from a psychosocial perspective.

In the Australian context, person-centred, nonpharmacological treatments for BPSD have received preliminary support from a recent randomized controlled trial (RCT), which examined the efficacy of a structured clinical protocol for managing challenging behaviours in patients with older-onset dementia [22]. This study found that compared to an active control condition involving staff training *alone*, staff training and concurrent clinical support were associated with sustained improvements in both staff coping and resident behaviour. These promising results suggest that compared to standalone staff training programs which are typically associated with mixed results [14], there appear to be measurable added benefits of providing concurrent clinical support in using a structured clinical protocol. Despite these promising findings, research is yet to evaluate the utility of this biopsychosocial model for treating BPSD in individuals with PNDs in whom BSPDs are often underrecognised and untreated over a typically protracted disease course.

**Figure 1:**
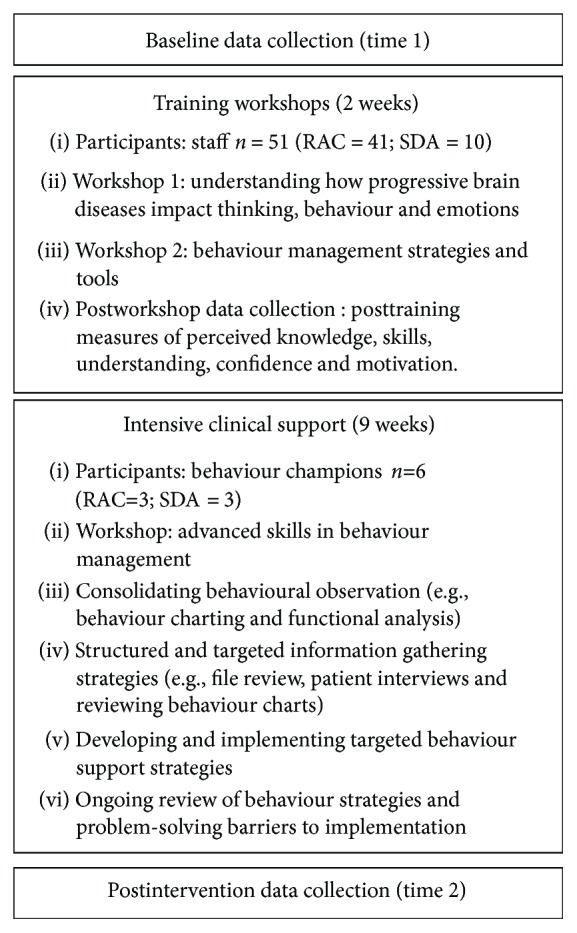
Timeline and protocol.

In addressing this substantial gap in knowledge, we developed a PND-specific structured clinical protocol that emphasised a person-centred, biopsychosocial approach targeting case-specific causal factors in BPSD in PNDs. In keeping with recent evidence that the benefits of staff training in BPSD clinical protocols are not maintained in the absence of clinical support [22], the three-tier training program involved (i) intensive staff training workshops delivered to all consenting facility staff; (ii) 9 weeks of intensive clinical support for a subgroup of facility staff (“behaviour champions”); and (iii) development and implementation of tailored procedures and resources intended to incorporate these principles into the organisational policy framework. “Behaviour champions” received intensive clinical supervision to implement the structured clinical protocol for residents living with PNDs. This intensive staff training program was grounded in the model for treatment of BPSD outlined by Brechin et al. [21], designed to empower staff with the skills and knowledge to more effectively manage the impact of BSPDs, and thus reduce reliance on external mental health services. A summary of the clinical protocol is provided in Figure 1, and further details are available from the corresponding author on request.

Due to the progressive and severely debilitating nature of PNDs, individuals with these conditions often require access to residential accommodation facilities that can provide high levels of specialist care and support. However, in the Australian context, facilities can differ markedly in terms of organisational culture and model of care, level of staff training and skills, and staff to resident ratios. Some individuals with PNDs may have access to specialist disability accommodation facilities tailored to high care needs of individuals under the age of 65. These specialist facilities typically benefit from smaller numbers of beds, higher staff to resident ratios, and an organisational ethos that promotes community participation and “living well.” Given the limited availability of specialist disability accommodation facilities, many people with PNDs reside in large residential aged care facilities (RAC), which predominantly cater for older adults (over the age of 65) with high dependency needs. These facilities generally have fewer residents with young-onset PNDs, and individuals with PNDs typically enter these facilities during the later stages of illness when their need for physical, social, and emotional support is greatest [23].

Since different residential accommodation settings present distinct challenges and opportunities, this study aimed to evaluate the utility of the structured clinical protocol in both RAC and SDA. Since intensive training and clinical support would offer the substantial input required to change the clinical culture of the RAC, we hypothesised that the intervention would be associated with significant reductions in resident BPSD and improvements in staff coping, in both the RAC and SDA. The primary outcome measure was changes in resident BPSD, and the secondary outcome measure was changes in staff coping.

## 2. Method

### 2.1. Participants

#### 2.1.1. Residents

Residents were recruited from a residential aged care (RAC) facility and a specialist disability accommodation (SDA) facility located in metropolitan Melbourne, Australia. The RAC comprised 45 beds (including 15 PND-specific beds) and 62 direct care staff. The SDA comprised a 6-bed, PND-specific facility employing 10 direct care staff.

At both facilities, all residents with a diagnosis of PND were approached to participate in the study. Residents provided informed consent after receiving information packs detailing the study. Accounting for resident deaths or removal from facilities during the study, the final sample comprised 13 individuals, 7 residing in the RAC (*M* age = 58.29 years, SD = 13.74) and 6 in the SDA (*M* age = 49.83 years, SD = 5.49).

#### 2.1.2. Staff

Staff at each facility were recruited through project scoping sessions conducted by a registered clinical neuropsychologist. At project scoping sessions, staff were provided a verbal summary of the project goals and processes, as well as a comprehensive participant information and consent form. At these meetings, informed written consent was obtained from 51 staff members (RAC: *n* = 41; SDA: *n* = 10).

### 2.2. Materials

#### 2.2.1. Resident Demographic Data

Demographic data, including age, gender, time residing at the facility, PND diagnosis, and other relevant diagnoses, were obtained from the residents' medical files at preintervention baseline.

#### 2.2.2. Resident BPSD

The *Neuropsychiatric Inventory-Nursing Home Version (NPI-NH)* was used to assess the frequency of BPSD as rated by the behaviour champions. The NPI-NH is a modified version of the original Neuropsychiatric Inventory [24] designed to measure BPSD symptoms in geriatric patients. A structured interview format is used in both instruments to assess 12 areas of BPSD symptomatology commonly found in dementing illnesses (i.e., delusions, hallucinations, agitation/aggression, depression, anxiety, euphoria/elation, apathy/indifference, disinhibition, irritability/lability, aberrant motor behaviour, night-time behaviour, and appetite/eating changes).

Staff provide ratings of symptom frequency and severity, and rate the degree of organisational disruption (i.e., extra staff stress and workload) caused by the presenting symptoms. Frequency scores range from 0 to 4, and severity scores range from 0 to 3. A symptom subscale score is calculated from the product of the severity and the frequency scores for each domain, with scores ranging from 0 to 12. The sum of the subscale scores is calculated to form the total score, ranging from 0 to 144. High scores on each scale represent a higher level of behavioural symptoms. A major study on the validity of the NPI-NH has been published [25]. These researchers demonstrated that licensed vocational nurses provided more accurate ratings than certified nurses' aides, supporting the use of the patients' primary nurse (i.e., behaviour champion) in the present study.

#### 2.2.3. Staff Demographic and Outcome Measures

Demographic data, including age, gender, and years worked at the facility, were obtained at baseline from each participating staff member. Self-reported confidence, knowledge, motivation, absenteeism, and use of employee support programs were measured using an in-house questionnaire.

Job strain, not related to specific residents, was measured using the 27-item *Strain in Dementia Care Scale (SDCS)* [7]. This measure was developed specifically for use in dementia care settings. The SDCS contains items relating to frustrated empathy, difficulty in understanding residents, balancing competing needs, balancing emotional involvement with residents, and perceived lack of appreciation from others. Staff rated how frequently a situation or feeling related to care of residents was experienced, from 1 (never/rarely) to 4 (very often), and how much stress the situation or feeling caused when it did occur, from 1 (none/hardly any) to 4 (high stress). A total score was calculated for frequency of strains and stress associated with items above. Internal reliability was high in this study, at *a* = 0.88 for the total frequency score and *a* = 0.93 for the total stress score.

All staff outcome measures were completed at preintervention baseline (T1) and immediately following the 11-week intervention program (T2).

### 2.3. Procedure

Ethics approval for the study was obtained from Calvary Health Care Bethlehem (CHCB) Human Research Ethics Committee and Melbourne Health Human Research Ethics Committee.

We conducted a pilot study to evaluate the utility of a structured clinical protocol for reducing the impact of BPSDs in residents living with PND. Data was collected at preintervention baseline, pre- and postworkshop training, and immediately following the roll-out of the entire 11-week intervention package which involved facility-wide staff workshop training (2 weeks) and an advanced training program involving a workshop and clinical supervision/support to a smaller subset of “behaviour champions” (9 weeks). The intervention protocol is described in the sections that follow and summarised in Figure 1.

#### 2.3.1. Recruitment

Residents were recruited through consultation with senior staff at both facilities. The inclusion criteria required a positive diagnosis of progressive neurological.

#### 2.3.2. Staff Training Workshops

All enrolled staff (*n* = 51) attended two ninety-minute training workshop sessions, which covered core components of the structured clinical protocol. Pre- and posttraining self-report questionnaires were administered to all staff that attended training sessions. Using a four-point Likert scale (1 = none to 4 = excellent), staff rated their perceived knowledge, understanding, skills, confidence, and motivation to engage in person centred, context-sensitive behaviour management approaches.

#### 2.3.3. Intensive Clinical Support

A clinical neuropsychologist experienced in working with BPSD provided an additional 9-week advanced training package for behaviour champions enrolled at each site. The program involved a 90-minute advanced training workshop (week 1), followed by clinical support provided over eight weeks, with supervision provided weekly for the first month and fortnightly for the second month. Clinical supervision sessions focused on assisting staff in implementing the structured clinical protocol to manage resident BPSD (see Figure 1).

### 2.4. Data Analysis

All data were analysed using IBM SPSS 22.0 and screened for violations of normality.

Normality plots indicated that primary and secondary outcome measures (NPI-NH and SDCS, resp.) were normally distributed, and preliminary analysis indicated no violation of assumptions across analyses unless otherwise stated.

Site comparisons (RAC versus SDA) were conducted using independent samples *t*-tests for the following descriptive variables: resident age, baseline BPSD, staff age, and prior training. Chi-square analysis was conducted to determine whether there was sex differences in residents and staff between sites. Percentages and frequencies are reported for the following characteristics: prior training (yes/no). For primary and secondary outcome analyses, repeated measures *t*-tests were conducted for each outcome measure (baseline T1 scores–postintervention T2 scores) at both sites.

## 3. Results

### 3.1. Resident Characteristics

Descriptive statistics for the resident sample are provided in Table 1. Univariate analyses revealed that the RAC and SDA did not significantly differ on baseline total NPI-NH scores (*t*(1, 11) = 1.07, *p* = 0.307), such that residents at each facility had comparable levels of BPSD at baseline. No significant differences were identified for age or gender.

### 3.2. Staff Characteristics

#### 3.2.1. Demographics by Site

Descriptive statistics for the staff sample are provided in Table 2. Groups differed in number of years worked in the facility, such that RAC staff reported longer tenure at the facility than those at the SDA. However, this difference did not reach statistical significance (*p* = 0.078).

#### 3.2.2. Staff Perceptions of Self and Organisational Efficacy by Site

Table 2 presents baseline measures of staff self- and organisational efficacy, as assessed by staff ratings of their own knowledge, skills, confidence, and motivation to manage BPSD, in addition to perceptions of organisational efficacy in addressing BPSD. Staff at the RAC endorsed lower levels of motivation to manage BPSD (*M* = 3.46; SD = 1.31) than SDA staff (*M* = 4.20; SD = 0.79); however, the difference did not reach statistical significance (*p* = 0.095). Both groups were comparable on other measures of self-efficacy including baseline perceptions of knowledge/skills, understanding of policies/procedure, confidence, and support.

Analysis of staff perceptions of organisational efficacy revealed significant group differences, such that RAC staff endorsed lower levels of support from family carers of residents (*p* = 0.023). RAC staff also endorsed lower levels of organisational success in reducing distress in residents with BPSD; however, this difference did not reach statistical significance (*p* = 0.054).

### 3.3. Workshop Training Outcomes by Site

#### 3.3.1. Workshop 1: Understanding How Progressive Brain Diseases Impact Thinking, Behaviour, and Emotions

For RAC, paired *t*-tests revealed significant posttraining increases on measures of staff perceived knowledge (*p* < 0.001), skills (*p* = 0.038), understanding (*p* = 0.001), confidence (*p* = 0.008), and motivation (*p* = 0.040) to identify and manage cognitive, behavioural, and emotional symptoms in people with progressive neurological diseases. Similarly, staff at the SDA reported significant posttraining increases in knowledge (*p* = 0.015), skills (*p* = 0.040), and motivation (*p* = 0.041) in this area of competence. There were no significant changes observed in staff ratings of confidence at the SDA.

#### 3.3.2. Workshop 2: Behaviour Management Strategies and Tools

For RAC, paired *t*-tests revealed significant posttraining increases on measures of staff perceived knowledge (*p* = 0.002), skills (*p* = 0.002), understanding (*p* < 0.001), and motivation (*p* = 0.001) in completing behaviour charts and implementing behaviour support plans. Similarly, staff at the SDA reported significant posttraining increases in knowledge (*p* = 0.035); however, increases in perceived skills did not reach statistical significance (*p* = 0.074).

### 3.4. Outcomes by Site: BPSD Ratings

#### 3.4.1. RAC

As shown in Table 3, for NPI-NH ratings, there was no significant time effect for BSPSDs from T1 to T2 (*t*(1, 6) = 1.18, *p* = 0.283, *d* = 0.33). Similarly, there was no significant reduction in perceived level of organisational disruption associated with the behaviours (*t*(1, 6) = 0.536, *p* = 0.611, *d* = 0.13).

#### 3.4.2. SDA

Analyses of NPI-NH ratings revealed a significant reduction in BPSD from T1 to T2 (*t*(1, 5) = 2.58, *p* = 0.049, *d* = 0.84). Reductions were also observed in perceived levels of organisational disruption associated with the behaviours; however, this change did not reach statistical significance (*t*(1, 5) = 1.97, *p* = 0.108, *d* = 1.13).

### 3.5. Outcomes by Site: Staff Coping

#### 3.5.1. RAC

Longitudinal analyses of the Strain in Dementia Care Scale (SDCS) ratings included follow-up data collected from 25 of the 41 staff (61%) initially enrolled at T1. Analyses of demographic and psychological characteristics of participating versus nonparticipating staff at T2 revealed no significant group differences in age, gender, or ratings of motivation, skills, or knowledge as assessed at T1.

As shown in Table 4, no significant time effect was found on the total frequency (of perceived job strains) scale (*t*(1, 24) = 1.56, *p* = 0.131, *d* = 0.32). Though reductions in total stress (associated with perceived job strains) did not reach statistical significance (*t*(1, 24) = 1.77, *p* = 0.090), a medium effect size was observed (*d* = 0.50).

#### 3.5.2. SDA

Longitudinal analyses of the Strain in Dementia Care Scale (SDCS) ratings included follow-up data collected from 7 of the 10 staff (70%) initially enrolled at T1. Paired *t*-tests revealed no significant time effect on the total frequency (of perceived job strains) scale (*t*(1, 6) = −0.17, *p* = 0.872, *d* = 0.12). In contrast, there was a statistically significant reduction in total stress (associated with job strains) from T1 to T2 (*t*(1, 6) = 2.66, *p* = 0.045, *d* = 0.85).

### 3.6. Additional Analyses by Diagnostic Group

To account for potential variation in the behavioural profile and disease progression of MS and non-MS residents, data were reanalysed after excluding the non-MS residents. We found that exclusion of these residents did not significantly alter the results on the primary NPI-NH outcome measure.

## 4. Discussion

To our knowledge, this is the first study of its kind to evaluate the efficacy of training and clinical support in implementing a structured clinical protocol to reduce the impact of behavioural and psychological symptoms of dementia (BPSD) in progressive neurological diseases (PNDs). Due to the progressive and severely debilitating nature of PNDs, individuals with these conditions frequently require access to residential accommodation facilities that can provide high levels of care and support. In the Australian context, specialist disability accommodation facilities are limited, and when access is not available, these patients are typically placed into general residential aged care facilities, which often differ markedly in terms of staff expertise, ethos, and model of care. To account for these differences and ensure the generalisability of findings, we aimed to evaluate the feasibility and efficacy of the structured clinical protocol in both residential aged care (RAC) and specialist disability accommodation (SDA).

Grounded in Brechin et al.'s [21] model of treatment for BPSD, which emphasises a proactive, person-centred approach to identifying and targeting contextual environmental causes and risks for BPSD, we expected that the combination of training plus intensive clinical support would offer the substantial input required to change clinical culture in both types of facilities. We therefore hypothesised that the intervention would be associated with significant reductions in BPSD and improvement in staff coping, in both the RAC and SDA.

Results partially support our expectations. While findings provide preliminary support for the feasibility and efficacy of a PND-specific structured clinical protocol, we found that outcomes markedly differed between intervention settings. Encouragingly, and in keeping with expectations, we found that in the SDA setting, the 11-week program was associated with statistically significant reductions in BPSD. Findings also revealed significant improvements in staff coping in the SDA setting, as indicated by significant reductions in total job-related stress from T1 to T2. Contrary to expectations, training and intensive clinical support were not associated with a reduction in BPSD and staff stress at the RAC. Interestingly, while there was no effect of time on BPSD, reductions in job-related stress approached statistical significance.

While the significant effect of staff training and intensive clinical support was found only in the SDA setting, these results from a small sample of individuals with PNDs have important clinical and theoretical significance. Firstly, these findings add to an emerging body of research supporting the efficacy of nonpharmacological approaches to management of BPSD [21, 22]. Since existing research in this area has been limited only to a single RCT of a structured clinical protocol for BPSD in individuals with older-onset dementia of the Alzheimer's type [22], our findings extend on this earlier work to suggest that this intervention model may be an acceptable and efficacious approach to management of BPSD associated with young-onset PNDs, at least in the SDA setting.

From a theoretical standpoint, these findings suggest that BPSD are amenable to change via context-sensitive, behaviourally focused approaches that aim to respond to the individual's unmet psychological, social, and emotional needs [11, 21]. As such, based on this data, it seems reasonable to infer that when delivered in isolation, pharmacological approaches are likely insufficient to address the multiple aetiologies for BPSD in PNDs, including genetic, psychosocial, neurobiological, medical, and physical factors, which interact dynamically to give rise to a complex constellation of symptoms that are unique to the individual [3, 21, 22]. Current findings are also in keeping with recent international guidelines (e.g., International Psychogeriatric Association Complete Guide to BPSD, 2012), which suggest that nonpharmacological treatments may be useful to integrate into usual care for people with PNDs.

In keeping with expectations, significant decreases in staff job stress at the SDA were observed alongside significant reductions in resident BPSD. Though the precise mechanism underlying changes in staff job stress is uncertain, we speculate that reductions in staff stress were mediated by changes in staff perceptions of resident behaviour [22]. Moreover, in keeping with previous theoretical and empirical links between staff stress and resident BPSD [8, 9], results show that PND-specific training and clinical support are associated with measurable reductions in staff stress, which may in turn increase staff capacity to identify indicators of resident distress and respond accordingly [26].

While perhaps surprising, limited support for effects of the structured clinical protocol on BPSD in the RAC setting represents an important finding. While the absence of a statistically significant effect on BPSD may be at least partly related to the possibility that staff stress levels did not significantly change in the RAC [8, 26], site variation in BPSD outcomes should also be considered in light of organisational and workforce level barriers that may be unique to the RAC context. Firstly, due to the standardized nature of the clinical protocol and constraints on training resources, the number of staff receiving intensive clinical support (i.e., “behaviour champions”) was limited to three per intervention setting. Since the RAC was a substantially larger facility both in terms of total staff and residents, the behaviour champion to staff ratio was substantially lower than in the SDA setting. Specifically, compared to the SDA setting in which the ratio of behaviour champions to staff was 1 : 3, the ratio of 1 : 15 in the RAC setting conferred considerably less opportunities for facility staff to observe and model behavioural support skills learnt by the behaviour champions. Similarly, compared to SDA where the ratio of behaviour champions to residents was 1 : 2, the lower behaviour champion to resident ratio in the RAC (i.e., 1 : 15) likely contributed to reduced opportunities for behaviour champions to deliver individualised behaviour support and facilitate a consistent approach to care. Taken together with the lower behaviour champion to staff ratio, the null results in the RAC are perhaps not surprising and underscore a need to consider these factors when implementing future RCTs in this population.

Secondly, to the best of our knowledge, this study represents the first to systematically measure baseline staff psychological characteristics, which may be another factor contributing to the observed differences in outcomes between sites. Interestingly, staff in RAC endorsed lower levels of motivation to engage in BPSD management at baseline. Though the correlates of reduced staff motivation cannot be established, we speculate that reduced staff motivation may be intimately associated with the organisational ethos of RAC, which focuses primarily on alleviating suffering and supporting the end of one's life [27], rather than engaging in more active management or neuro-rehabilitation approaches [23]. As such, in a residential facility where roles, policies, and procedures are situated within a more task-oriented framework, it is perhaps not surprising that staff would assign lesser value and endorse reduced motivation to engage in proactive, person-centred behaviour management approaches. In contrast, it is noteworthy that statistically significant effects were observed in the setting, where individually tailored, context-sensitive interventions are likely considered to align more closely to an organisational ethos centred on residents “living well” and retaining independence, where staff are encouraged to support resident participation and independence in daily activities.

Although speculative, the potential impact of these factors should prompt researchers to consider the influence of an organisation's clinical culture on intervention outcomes. To systematically address these factors in future research, targeted strategies and resources are needed to embed these principles into organisational procedures, thereby increasing the likelihood that staff will reconceptualise their clinical role and view behaviour management as a core component of their daily tasks and duties. Accordingly, adaptations to the current clinical protocol might involve embedding BSPD management principles into the general care regime at both intake and clinical handover (i.e., dedicated behaviour management component of clinical handover and mandatory behaviour charting). Based on anecdotal evidence from the current study, the success of these strategies will likely require top-down support from senior managers and staff who have capacity to lead, model, and embed these systemic changes into the general care regime. The allocation of appropriate resources (e.g., incentives/organisational recognition and higher staff : resident ratios) would also be required to make these feasible recommendations. These observations are in keeping with meta-analytic evidence that supportive workplace relations (e.g., support and incentive-based programs) are a key factor underlying the successful translation of skills into practice [28].

The current study did have several limitations. Since resource limitations precluded the possibility of extended follow-up, further studies are required to establish whether the observed gains are maintained over time. Furthermore, longitudinal studies of this nature are prone to sample attrition, reflected in the T2 follow-up rate of staff enrolled in the study. Despite this weakness, we collected detailed information on staff demographic and psychological variables, analyses of which revealed no significant differences between participating and nonparticipating groups at T2 follow-up.

A further caveat pertains to the intensity and duration of clinical support for behaviour champions. For instance, anecdotal evidence suggests that an extended period of clinical support (e.g., 12 months) with increased time between supervision sessions (e.g., monthly versus weekly sessions) may have been beneficial and provided additional scope to embed the structured clinical protocol into organisational policy and clinical procedures; an approach that would likely contribute to a more robust shift in organisational clinical culture.

Finally, since a large proportion of our sample comprised individuals living with multiple sclerosis (MS), the generalizability of our findings to lower prevalence PNDs (e.g., MND and PD) is somewhat limited. To account for potential variation in the behavioural profile and disease progression of MS and non-MS residents, data were reanalysed after excluding the non-MS residents. We found that exclusion of these residents did not significantly alter the results on any of the primary outcome measures. It is also important to note that despite the large proportion of residents with MS in this study, the composition of our sample is consistent with the higher population prevalence of multiple sclerosis, particularly among those living in residential care facilities housing individuals under the age of 65 years.

Despite the limitations of the study and the notable absence of significant reductions in BPSD at the RAC setting, it is noteworthy that training in the RAC was associated with significant increases in staff knowledge, skills, and confidence from pre- to posttraining. Taken together with evidence that reductions in staff ratings of job-related stress approached statistical significance (with a medium-to-large effect size), findings are in keeping with evidence linking increased knowledge, skills, and support to reduced likelihood of stress reactions and staff burnout, which may in turn have important implications for the frequency and severity of resident BPSD [6, 8].

## 5. Conclusions

To our knowledge, this study represents the first to evaluate the feasability and efficacy of a structured clinical protocol for managing BPSD in PNDs across two distinct residential accommodation settings. Our results suggest that PND-specific training and clinical support in patient-centred care are associated with benefits for both residents and staff in SDA. Importantly, while we found that the intervention was linked to significant reductions in staff job stress and resident BPSD in the SDA setting, these effects were not observed in the RAC. While the nonsignificant reduction in staff job stress may at least partly explain the null effect of the structured clinical protocol on BPSD in the RAC, site variation in BPSD outcomes may relate to organisational and workforce level barriers that may be unique to the RAC context and should be systematically addressed in future RCT studies of larger PND samples.

## Figures and Tables

**Table 1:** Demographic and behavioural data for PND resident sample.

	Intervention setting		
	RAC	SDA	*p* value
Number of residents enrolled	7	6	—
Age (SD)	58.29 (13.74)	49.83 (5.49)	0.187
Females, *n* (%)	5 (71)	6 (100)	0.462
*PND diagnosis*, *n* (%)			
Multiple sclerosis	5 (72)	6 (100)	—
Huntington's disease	1 (14)	—	—
Parkinson's disease	1 (14)	—	—
Baseline total NPI	19.57 (9.18)	25.67 (11.34)	0.307
Baseline NPI disruption	7.86 (4.26)	8.67 (5.09)	0.760

**Table 2:** Demographic and psychological characteristics of participating staff.

	RAC	SDA	*p* value
*Demographics*			
*N*	41	10	—
Age, *M* (SD)	46.24 (12.55)	43.70 (9.43)	0.552
Tenure (years), *M* (SD)	8.58 (8.30)	3.77 (2.52)	0.078
Days absent	0.73 (2.54)	0.80 (1.32)	0.935
*Baseline perceptions*			
*BPSD self-efficacy total*∗	19.07 (4.08)	19.70 (1.77)	0.639
Training provided	2.95 (0.92)	2.80 (0.63)	0.626
Skills/knowledge	3.10 (0.77)	2.80 (0.63)	0.263
Policy/procedures	3.07 (1.19)	3.20 (0.63)	0.747
Confidence	3.17 (0.70)	3.30 (0.82)	0.616
Motivation	3.46 (1.31)	4.20 (0.79)	0.095
Support	3.32 (0.82)	3.40 (1.17)	0.794
*BPSD organisational efficacy total*∗	11.20 (2.74)	13.00 (2.83)	0.069
Reducing frequency	3.00 (0.89)	3.20 (0.63)	0.509
Research driven	2.63 (0.92)	3.00 (1.33)	0.307
Reducing distress	2.93 (0.79)	3.50 (0.97)	0.054
Supported by family/carers	2.63 (0.83)	3.30 (0.68)	0.023

∗ denotes the total score (composite measure) derived from subscales assessing perceived self and organisational efficacy in managing BPSDs.

**Table 3:** Means, standard deviations, and effect sizes on the NPI-NH.

	T1 baseline *M* (SD)	T2 follow-up *M* (SD)	Effect size *d*	Sig T1-T2
*RAC* (*n* = 7)				
NPI total	19.57 (9.18)	16.71 (7.87)	0.33	0.283
NPI disrupt	7.86 (4.26)	7.29 (4.35)	0.13	0.611
*SDA* (*n* = 6)				
NPI total	25.67 (11.34)	16.33 (10.78)	0.84	0.049
NPI disrupt	8.67 (5.09)	4.50 (1.05)	1.13	0.108

**Table 4:** Means, standard deviations, and effect sizes on the SDCS.

Facility	T1 baseline, *M* (SD)	T2 follow-up, *M* (SD)	Effect size *d*	Sig T1-T2
*RAC*				
Frequency of strains	58.80 (7.50)	56.56 (6.27)	0.32	0.131
Total stress	54.04 (12.48)	47.96 (11.64)	0.50	0.090
*SDA*				
Frequency of strains	48.00 (6.03)	48.67 (5.54)	0.12	0.872
Total stress	46.00 (10.00)	38.33 (7.99)	0.85	0.045
